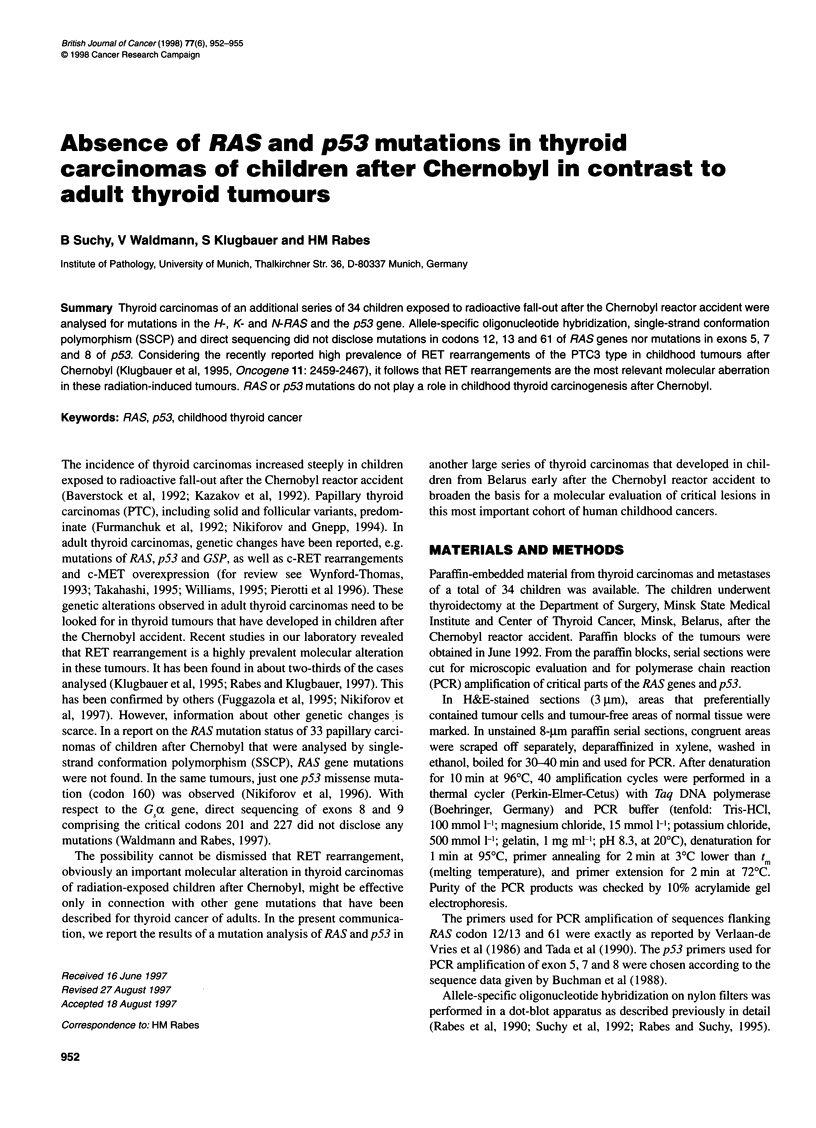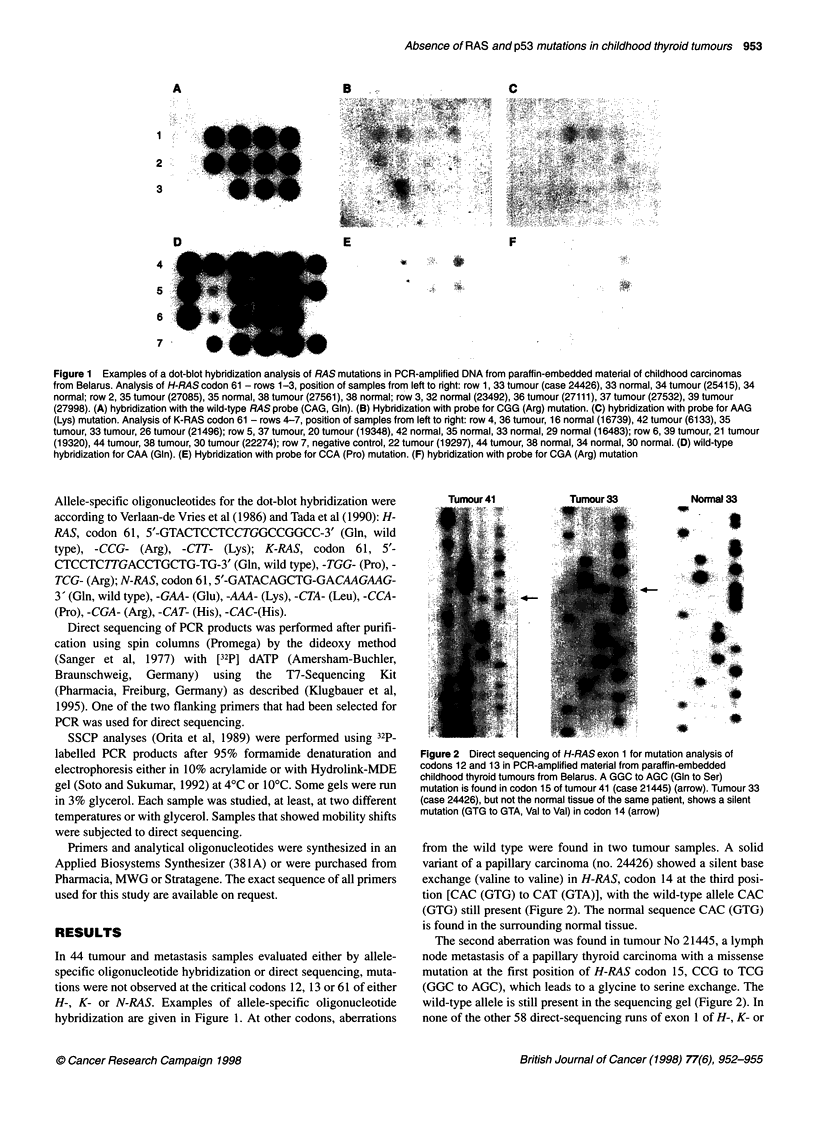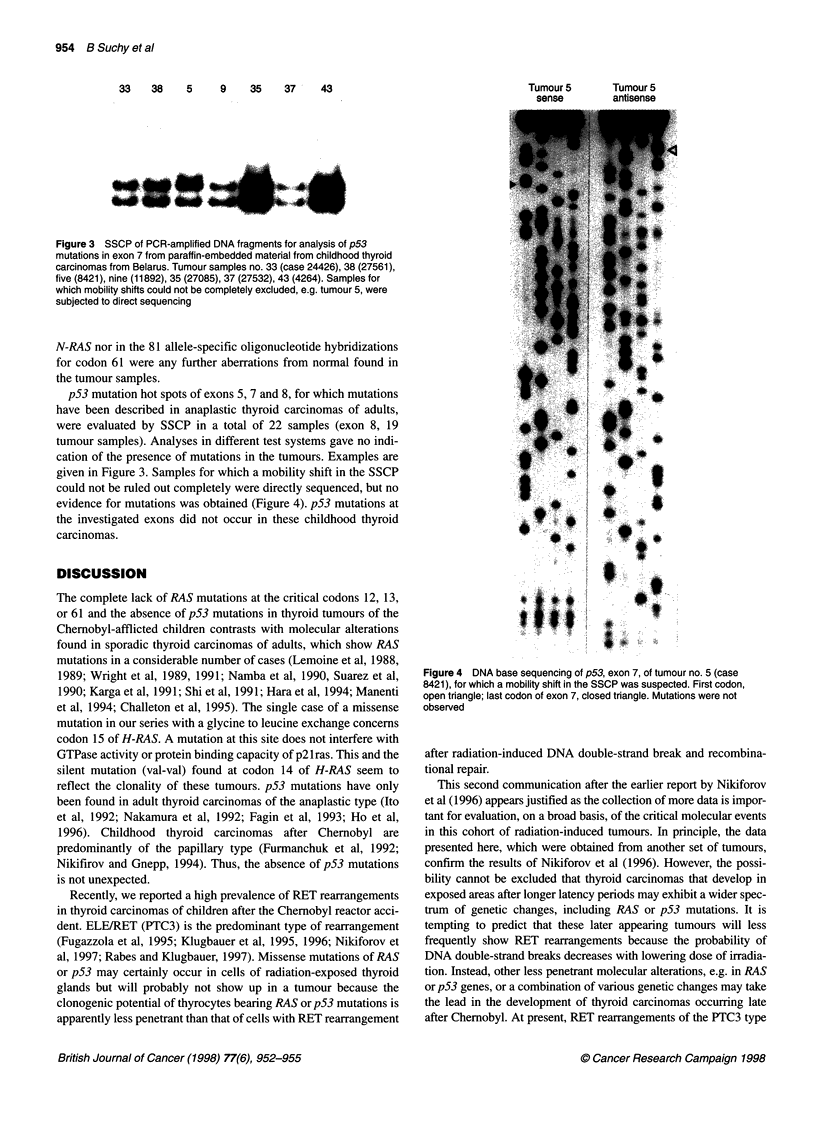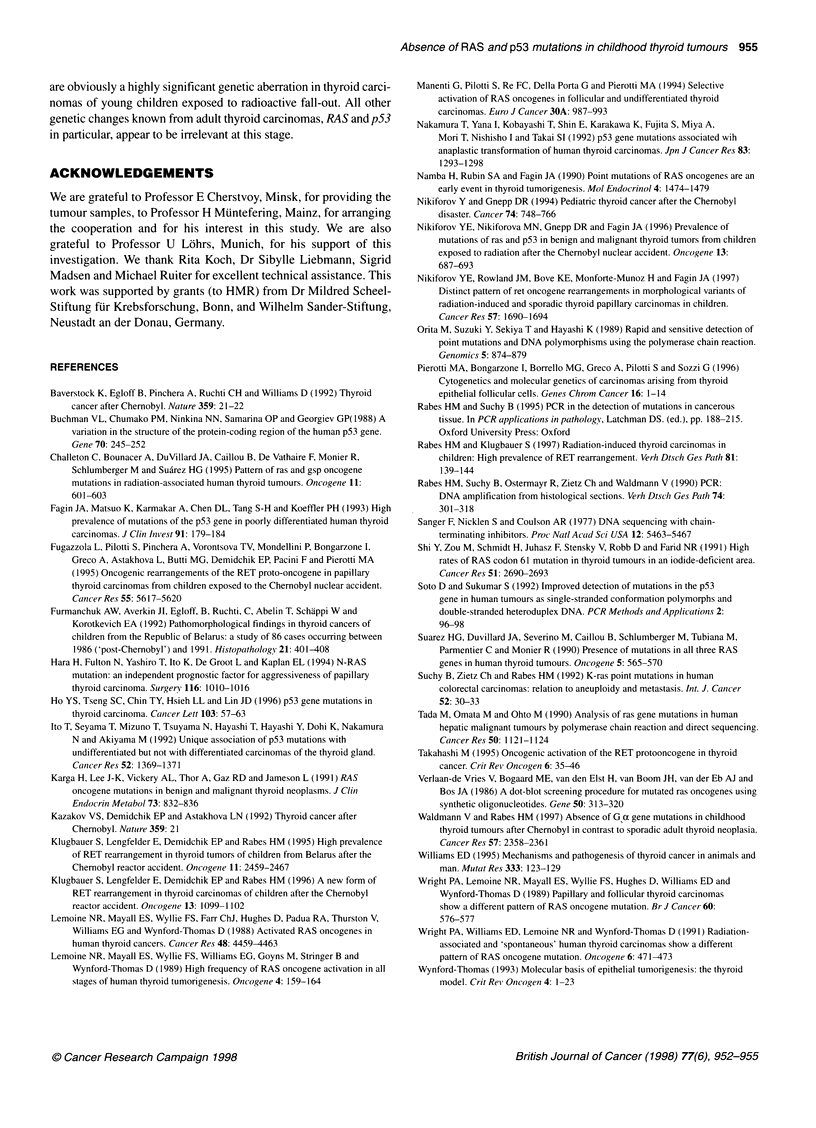# Absence of RAS and p53 mutations in thyroid carcinomas of children after Chernobyl in contrast to adult thyroid tumours.

**DOI:** 10.1038/bjc.1998.157

**Published:** 1998-03

**Authors:** B. Suchy, V. Waldmann, S. Klugbauer, H. M. Rabes

**Affiliations:** Institute of Pathology, University of Munich, Germany.

## Abstract

**Images:**


British Journal of Cancer (1998) 77(6), 952–955
© 1998 Cancer Research Campaign

### Absence of *RAS* and *p53* mutations in thyroid carcinomas of children after Chernobyl in contrast to adult thyroid tumours

**B Suchy, V Waldmann, S Klugbauer and HM Rabes**

Institute of Pathology, University of Munich, Thalkirchner Str. 36, D-80337 Munich, Germany

**Summary** Thyroid carcinomas of an additional series of 34 children exposed to radioactive fall-out after the Chernobyl reactor accident were analysed for mutations in the *H-*, *K-* and *N-RAS* and the *p53* gene. Allele-specific oligonucleotide hybridization, single-strand conformation polymorphism (SSCP) and direct sequencing did not disclose mutations in codons 12, 13 and 61 of *RAS* genes nor mutations in exons 5, 7 and 8 of *p53*. Considering the recently reported high prevalence of RET rearrangements of the PTC3 type in childhood tumours after Chernobyl (Klugbauer et al, 1995, *Oncogene* **11**: 2459-2467), it follows that RET rearrangements are the most relevant molecular aberration in these radiation-induced tumours. *RAS* or *p53* mutations do not play a role in childhood thyroid carcinogenesis after Chernobyl.

**Keywords:** *RAS*, *p53*, childhood thyroid cancer

The incidence of thyroid carcinomas increased steeply in children exposed to radioactive fall-out after the Chernobyl reactor accident (Baverstock et al, 1992; Kazakov et al, 1992). Papillary thyroid carcinomas (PTC), including solid and follicular variants, predominate (Furmanchuk et al, 1992; Nikiforov and Gnepp, 1994). In adult thyroid carcinomas, genetic changes have been reported, e.g. mutations of *RAS*, *p53* and *GSP*, as well as c-RET rearrangements and c-MET overexpression (for review see Wynford-Thomas, 1993; Takahashi, 1995; Williams, 1995; Pierotti et al 1996). These genetic alterations observed in adult thyroid carcinomas need to be looked for in thyroid tumours that have developed in children after the Chernobyl accident. Recent studies in our laboratory revealed that RET rearrangement is a highly prevalent molecular alteration in these tumours. It has been found in about two-thirds of the cases analysed (Klugbauer et al, 1995; Rabes and Klugbauer, 1997). This has been confirmed by others (Fuggazola et al, 1995; Nikiforov et al, 1997). However, information about other genetic changes is scarce. In a report on the *RAS* mutation status of 33 papillary carcinomas of children after Chernobyl that were analysed by single-strand conformation polymorphism (SSCP), *RAS* gene mutations were not found. In the same tumours, just one *p53* missense mutation (codon 160) was observed (Nikiforov et al, 1996). With respect to the $G_s\alpha$ gene, direct sequencing of exons 8 and 9 comprising the critical codons 201 and 227 did not disclose any mutations (Waldmann and Rabes, 1997).

The possibility cannot be dismissed that RET rearrangement, obviously an important molecular alteration in thyroid carcinomas of radiation-exposed children after Chernobyl, might be effective only in connection with other gene mutations that have been described for thyroid cancer of adults. In the present communication, we report the results of a mutation analysis of *RAS* and *p53* in another large series of thyroid carcinomas that developed in children from Belarus early after the Chernobyl reactor accident to broaden the basis for a molecular evaluation of critical lesions in this most important cohort of human childhood cancers.

*Received 16 June 1997*
*Revised 27 August 1997*
*Accepted 18 August 1997*

*Correspondence to:* HM Rabes

### MATERIALS AND METHODS

Paraffin-embedded material from thyroid carcinomas and metastases of a total of 34 children was available. The children underwent thyroidectomy at the Department of Surgery, Minsk State Medical Institute and Center of Thyroid Cancer, Minsk, Belarus, after the Chernobyl reactor accident. Paraffin blocks of the tumours were obtained in June 1992. From the paraffin blocks, serial sections were cut for microscopic evaluation and for polymerase chain reaction (PCR) amplification of critical parts of the *RAS* genes and *p53*.

In H&E-stained sections (3 μm), areas that preferentially contained tumour cells and tumour-free areas of normal tissue were marked. In unstained 8-μm paraffin serial sections, congruent areas were scraped off separately, deparaffinized in xylene, washed in ethanol, boiled for 30–40 min and used for PCR. After denaturation for 10 min at 96°C, 40 amplification cycles were performed in a thermal cycler (Perkin-Elmer-Cetus) with *Taq* DNA polymerase (Boehringer, Germany) and PCR buffer (tenfold: Tris-HCl, 100 mmol $l^{-1}$; magnesium chloride, 15 mmol $l^{-1}$; potassium chloride, 500 mmol $l^{-1}$; gelatin, 1 mg $ml^{-1}$; pH 8.3, at 20°C), denaturation for 1 min at 95°C, primer annealing for 2 min at 3°C lower than $t_m$ (melting temperature), and primer extension for 2 min at 72°C. Purity of the PCR products was checked by 10% acrylamide gel electrophoresis.

The primers used for PCR amplification of sequences flanking *RAS* codon 12/13 and 61 were exactly as reported by Verlaan-de Vries et al (1986) and Tada et al (1990). The *p53* primers used for PCR amplification of exon 5, 7 and 8 were chosen according to the sequence data given by Buchman et al (1988).

Allele-specific oligonucleotide hybridization on nylon filters was performed in a dot-blot apparatus as described previously in detail (Rabes et al, 1990; Suchy et al, 1992; Rabes and Suchy, 1995).

**Figure 1** Examples of a dot-blot hybridization analysis of *RAS* mutations in PCR-amplified DNA from paraffin-embedded material of childhood carcinomas from Belarus. Analysis of *H-RAS* codon 61 – rows 1–3, position of samples from left to right: row 1, 33 tumour (case 24426), 33 normal, 34 tumour (25415), 34 normal; row 2, 35 tumour (27085), 35 normal, 38 tumour (27561), 38 normal; row 3, 32 normal (23492), 36 tumour (27111), 37 tumour (27532), 39 tumour (27998). (**A**) hybridization with the wild-type *RAS* probe (CAG, Gln). (**B**) Hybridization with probe for CGG (Arg) mutation. (**C**) hybridization with probe for AAG (Lys) mutation. Analysis of K-RAS codon 61 – rows 4–7, position of samples from left to right: row 4, 36 tumour, 16 normal (16739), 42 tumour (6133), 35 tumour, 33 tumour, 26 tumour (21496); row 5, 37 tumour, 20 tumour (19348), 42 normal, 35 normal, 33 normal, 29 normal (16483); row 6, 39 tumour, 21 tumour (19320), 44 tumour, 38 tumour, 30 tumour (22274); row 7, negative control, 22 tumour (19297), 44 tumour, 38 normal, 34 normal, 30 normal. (**D**) wild-type hybridization for CAA (Gln). (**E**) Hybridization with probe for CCA (Pro) mutation. (**F**) hybridization with probe for CGA (Arg) mutation

Allele-specific oligonucleotides for the dot-blot hybridization were according to Verlaan-de Vries et al (1986) and Tada et al (1990): *H-RAS*, codon 61, 5′-GTACTCCTC*CTG*GCCGGCC-3′ (Gln, wild type), *-CCG-* (Arg), *-CTT-* (Lys); *K-RAS*, codon 61, 5′-CTCCTC*TTG*ACCTGCTG-TG-3′ (Gln, wild type), *-TGG-* (Pro), *-TCG-* (Arg); *N-RAS*, codon 61, 5′-GATACAGCTG-GA*CAAGAAG*-3′ (Gln, wild type), *-GAA-* (Glu), *-AAA-* (Lys), *-CTA-* (Leu), *-CCA-* (Pro), *-CGA-* (Arg), *-CAT-* (His), *-CAC-*(His).

Direct sequencing of PCR products was performed after purification using spin columns (Promega) by the dideoxy method (Sanger et al, 1977) with [$^{32}$P] dATP (Amersham-Buchler, Braunschweig, Germany) using the T7-Sequencing Kit (Pharmacia, Freiburg, Germany) as described (Klugbauer et al, 1995). One of the two flanking primers that had been selected for PCR was used for direct sequencing.

SSCP analyses (Orita et al, 1989) were performed using $^{32}$P-labelled PCR products after 95% formamide denaturation and electrophoresis either in 10% acrylamide or with Hydrolink-MDE gel (Soto and Sukumar, 1992) at 4°C or 10°C. Some gels were run in 3% glycerol. Each sample was studied, at least, at two different temperatures or with glycerol. Samples that showed mobility shifts were subjected to direct sequencing.

Primers and analytical oligonucleotides were synthesized in an Applied Biosystems Synthesizer (381A) or were purchased from Pharmacia, MWG or Stratagene. The exact sequence of all primers used for this study are available on request.

### RESULTS

In 44 tumour and metastasis samples evaluated either by allele-specific oligonucleotide hybridization or direct sequencing, mutations were not observed at the critical codons 12, 13 or 61 of either *H-*, *K-* or *N-RAS*. Examples of allele-specific oligonucleotide hybridization are given in Figure 1. At other codons, aberrations

**Figure 2** Direct sequencing of *H-RAS* exon 1 for mutation analysis of codons 12 and 13 in PCR-amplified material from paraffin-embedded childhood thyroid tumours from Belarus. A GGC to AGC (Gln to Ser) mutation is found in codon 15 of tumour 41 (case 21445) (arrow). Tumour 33 (case 24426), but not the normal tissue of the same patient, shows a silent mutation (GTG to GTA, Val to Val) in codon 14 (arrow)

from the wild type were found in two tumour samples. A solid variant of a papillary carcinoma (no. 24426) showed a silent base exchange (valine to valine) in *H-RAS*, codon 14 at the third position [CAC (GTG) to CAT (GTA)], with the wild-type allele CAC (GTG) still present (Figure 2). The normal sequence CAC (GTG) is found in the surrounding normal tissue.

The second aberration was found in tumour No 21445, a lymph node metastasis of a papillary thyroid carcinoma with a missense mutation at the first position of *H-RAS* codon 15, CCG to TCG (GGC to AGC), which leads to a glycine to serine exchange. The wild-type allele is still present in the sequencing gel (Figure 2). In none of the other 58 direct-sequencing runs of exon 1 of *H-*, *K-* or

**Figure 3** SSCP of PCR-amplified DNA fragments for analysis of *p53* mutations in exon 7 from paraffin-embedded material from childhood thyroid carcinomas from Belarus. Tumour samples no. 33 (case 24426), 38 (27561), five (8421), nine (11892), 35 (27085), 37 (27532), 43 (4264). Samples for which mobility shifts could not be completely excluded, e.g. tumour 5, were subjected to direct sequencing

*N-RAS* nor in the 81 allele-specific oligonucleotide hybridizations for codon 61 were any further aberrations from normal found in the tumour samples.

*p53* mutation hot spots of exons 5, 7 and 8, for which mutations have been described in anaplastic thyroid carcinomas of adults, were evaluated by SSCP in a total of 22 samples (exon 8, 19 tumour samples). Analyses in different test systems gave no indication of the presence of mutations in the tumours. Examples are given in Figure 3. Samples for which a mobility shift in the SSCP could not be ruled out completely were directly sequenced, but no evidence for mutations was obtained (Figure 4). *p53* mutations at the investigated exons did not occur in these childhood thyroid carcinomas.

### DISCUSSION

The complete lack of *RAS* mutations at the critical codons 12, 13, or 61 and the absence of *p53* mutations in thyroid tumours of the Chernobyl-afflicted children contrasts with molecular alterations found in sporadic thyroid carcinomas of adults, which show *RAS* mutations in a considerable number of cases (Lemoine et al, 1988, 1989; Wright et al, 1989, 1991; Namba et al, 1990, Suarez et al, 1990; Karga et al, 1991; Shi et al, 1991; Hara et al, 1994; Manenti et al, 1994; Challeton et al, 1995). The single case of a missense mutation in our series with a glycine to leucine exchange concerns codon 15 of *H-RAS*. A mutation at this site does not interfere with GTPase activity or protein binding capacity of p21ras. This and the silent mutation (val-val) found at codon 14 of *H-RAS* seem to reflect the clonality of these tumours. *p53* mutations have only been found in adult thyroid carcinomas of the anaplastic type (Ito et al, 1992; Nakamura et al, 1992; Fagin et al, 1993; Ho et al, 1996). Childhood thyroid carcinomas after Chernobyl are predominantly of the papillary type (Furmanchuk et al, 1992; Nikifirov and Gnepp, 1994). Thus, the absence of *p53* mutations is not unexpected.

Recently, we reported a high prevalence of RET rearrangements in thyroid carcinomas of children after the Chernobyl reactor accident. ELE/RET (PTC3) is the predominant type of rearrangement (Fugazzola et al, 1995; Klugbauer et al, 1995, 1996; Nikiforov et al, 1997; Rabes and Klugbauer, 1997). Missense mutations of *RAS* or *p53* may certainly occur in cells of radiation-exposed thyroid glands but will probably not show up in a tumour because the clonogenic potential of thyrocytes bearing *RAS* or *p53* mutations is apparently less penetrant than that of cells with RET rearrangement

**Figure 4** DNA base sequencing of *p53*, exon 7, of tumour no. 5 (case 8421), for which a mobility shift in the SSCP was suspected. First codon, open triangle; last codon of exon 7, closed triangle. Mutations were not observed after radiation-induced DNA double-strand break and recombinational repair.

This second communication after the earlier report by Nikiforov et al (1996) appears justified as the collection of more data is important for evaluation, on a broad basis, of the critical molecular events in this cohort of radiation-induced tumours. In principle, the data presented here, which were obtained from another set of tumours, confirm the results of Nikiforov et al (1996). However, the possibility cannot be excluded that thyroid carcinomas that develop in exposed areas after longer latency periods may exhibit a wider spectrum of genetic changes, including *RAS* or *p53* mutations. It is tempting to predict that these later appearing tumours will less frequently show RET rearrangements because the probability of DNA double-strand breaks decreases with lowering dose of irradiation. Instead, other less penetrant molecular alterations, e.g. in *RAS* or *p53* genes, or a combination of various genetic changes may take the lead in the development of thyroid carcinomas occurring late after Chernobyl. At present, RET rearrangements of the PTC3 type

© Cancer Research Campaign 1998

are obviously a highly significant genetic aberration in thyroid carcinomas of young children exposed to radioactive fall-out. All other genetic changes known from adult thyroid carcinomas, *RAS* and *p53* in particular, appear to be irrelevant at this stage.

### ACKNOWLEDGEMENTS

We are grateful to Professor E Cherstvoy, Minsk, for providing the tumour samples, to Professor H Müntefering, Mainz, for arranging the cooperation and for his interest in this study. We are also grateful to Professor U Löhrs, Munich, for his support of this investigation. We thank Rita Koch, Dr Sibylle Liebmann, Sigrid Madsen and Michael Ruiter for excellent technical assistance. This work was supported by grants (to HMR) from Dr Mildred Scheel-Stiftung für Krebsforschung, Bonn, and Wilhelm Sander-Stiftung, Neustadt an der Donau, Germany.

### REFERENCES

Baverstock K, Egloff B, Pinchera A, Ruchti CH and Williams D (1992) Thyroid cancer after Chernobyl. *Nature* **359**: 21–22

Buchman VL, Chumako PM, Ninkina NN, Samarina OP and Georgiev GP(1988) A variation in the structure of the protein-coding region of the human p53 gene. *Gene* **70**: 245–252

Challeton C, Bounacer A, DuVillard JA, Caillou B, De Vathaire F, Monier R, Schlumberger M and Suárez HG (1995) Pattern of ras and gsp oncogene mutations in radiation-associated human thyroid tumours. *Oncogene* **11**: 601–603

Fagin JA, Matsuo K, Karmakar A, Chen DL, Tang S-H and Koeffler PH (1993) High prevalence of mutations of the p53 gene in poorly differentiated human thyroid carcinomas. *J Clin Invest* **91**: 179–184

Fugazzola L, Pilotti S, Pinchera A, Vorontsova TV, Mondellini P, Bongarzone I, Greco A, Astakhova L, Butti MG, Demidchik EP, Pacini F and Pierotti MA (1995) Oncogenic rearrangements of the RET proto-oncogene in papillary thyroid carcinomas from children exposed to the Chernobyl nuclear accident. *Cancer Res* **55**: 5617–5620

Furmanchuk AW, Averkin JI, Egloff, B, Ruchti, C, Abelin T, Schäppi W and Korotkevich EA (1992) Pathomorphological findings in thyroid cancers of children from the Republic of Belarus: a study of 86 cases occurring between 1986 ('post-Chernobyl') and 1991. *Histopathology* **21**: 401–408

Hara H, Fulton N, Yashiro T, Ito K, De Groot L and Kaplan EL (1994) N-RAS mutation: an independent prognostic factor for aggressiveness of papillary thyroid carcinoma. *Surgery* **116**: 1010–1016

Ho YS, Tseng SC, Chin TY, Hsieh LL and Lin JD (1996) p53 gene mutations in thyroid carcinoma. *Cancer Lett* **103**: 57–63

Ito T, Seyama T, Mizuno T, Tsuyama N, Hayashi T, Hayashi Y, Dohi K, Nakamura N and Akiyama M (1992) Unique association of p53 mutations with undifferentiated but not with differentiated carcinomas of the thyroid gland. *Cancer Res* **52**: 1369–1371

Karga H, Lee J-K, Vickery AL, Thor A, Gaz RD and Jameson L (1991) *RAS* oncogene mutations in benign and malignant thyroid neoplasms. *J Clin Endocrin Metabol* **73**: 832–836

Kazakov VS, Demidchik EP and Astakhova LN (1992) Thyroid cancer after Chernobyl. *Nature* **359**: 21

Klugbauer S, Lengfelder E, Demidchik EP and Rabes HM (1995) High prevalence of RET rearrangement in thyroid tumors of children from Belarus after the Chernobyl reactor accident. *Oncogene* **11**: 2459–2467

Klugbauer S, Lengfelder E, Demidchik EP and Rabes HM (1996) A new form of RET rearrangement in thyroid carcinomas of children after the Chernobyl reactor accident. *Oncogene* **13**: 1099–1102

Lemoine NR, Mayall ES, Wyllie FS, Farr ChJ, Hughes D, Padua RA, Thurston V, Williams EG and Wynford-Thomas D (1988) Activated RAS oncogenes in human thyroid cancers. *Cancer Res* **48**: 4459–4463

Lemoine NR, Mayall ES, Wyllie FS, Williams EG, Goyns M, Stringer B and Wynford-Thomas D (1989) High frequency of RAS oncogene activation in all stages of human thyroid tumorigenesis. *Oncogene* **4**: 159–164

Manenti G, Pilotti S, Re FC, Della Porta G and Pierotti MA (1994) Selective activation of RAS oncogenes in follicular and undifferentiated thyroid carcinomas. *Euro J Cancer* **30A**: 987–993

Nakamura T, Yana I, Kobayashi T, Shin E, Karakawa K, Fujita S, Miya A, Mori T, Nishisho I and Takai SI (1992) p53 gene mutations associated wih anaplastic transformation of human thyroid carcinomas. *Jpn J Cancer Res* **83**: 1293–1298

Namba H, Rubin SA and Fagin JA (1990) Point mutations of RAS oncogenes are an early event in thyroid tumorigenesis. *Mol Endocrinol* **4**: 1474–1479

Nikiforov Y and Gnepp DR (1994) Pediatric thyroid cancer after the Chernobyl disaster. *Cancer* **74**: 748–766

Nikiforov YE, Nikiforova MN, Gnepp DR and Fagin JA (1996) Prevalence of mutations of ras and p53 in benign and malignant thyroid tumors from children exposed to radiation after the Chernobyl nuclear accident. *Oncogene* **13**: 687–693

Nikiforov YE, Rowland JM, Bove KE, Monforte-Munoz H and Fagin JA (1997) Distinct pattern of ret oncogene rearrangements in morphological variants of radiation-induced and sporadic thyroid papillary carcinomas in children. *Cancer Res* **57**: 1690–1694

Orita M, Suzuki Y, Sekiya T and Hayashi K (1989) Rapid and sensitive detection of point mutations and DNA polymorphisms using the polymerase chain reaction. *Genomics* **5**: 874–879

Pierotti MA, Bongarzone I, Borrello MG, Greco A, Pilotti S and Sozzi G (1996) Cytogenetics and molecular genetics of carcinomas arising from thyroid epithelial follicular cells. *Genes Chrom Cancer* **16**: 1–14

Rabes HM and Suchy B (1995) PCR in the detection of mutations in cancerous tissue. In *PCR applications in pathology*, Latchman DS. (ed.), pp. 188–215. Oxford University Press: Oxford

Rabes HM and Klugbauer S (1997) Radiation-induced thyroid carcinomas in children: High prevalence of RET rearrangement. *Verh Dtsch Ges Path* **81**: 139–144

Rabes HM, Suchy B, Ostermayr R, Zietz Ch and Waldmann V (1990) PCR: DNA amplification from histological sections. *Verh Dtsch Ges Path* **74**: 301–318

Sanger F, Nicklen S and Coulson AR (1977) DNA sequencing with chain-terminating inhibitors. *Proc Natl Acad Sci USA* **12**: 5463–5467

Shi Y, Zou M, Schmidt H, Juhasz F, Stensky V, Robb D and Farid NR (1991) High rates of RAS codon 61 mutation in thyroid tumours in an iodide-deficient area. *Cancer Res* **51**: 2690–2693

Soto D and Sukumar S (1992) Improved detection of mutations in the p53 gene in human tumours as single-stranded conformation polymorphs and double-stranded heteroduplex DNA. *PCR Methods and Applications* **2**: 96–98

Suarez HG, Duvillard JA, Severino M, Caillou B, Schlumberger M, Tubiana M, Parmentier C and Monier R (1990) Presence of mutations in all three RAS genes in human thyroid tumours. *Oncogene* **5**: 565–570

Suchy B, Zietz Ch and Rabes HM (1992) K-ras point mutations in human colorectal carcinomas: relation to aneuploidy and metastasis. *Int. J. Cancer* **52**: 30–33

Tada M, Omata M and Ohto M (1990) Analysis of ras gene mutations in human hepatic malignant tumours by polymerase chain reaction and direct sequencing. *Cancer Res* **50**: 1121–1124

Takahashi M (1995) Oncogenic activation of the RET protooncogene in thyroid cancer. *Crit Rev Oncogen* **6**: 35–46

Verlaan-de Vries V, Bogaard ME, van den Elst H, van Boom JH, van der Eb AJ and Bos JA (1986) A dot-blot screening procedure for mutated ras oncogenes using synthetic oligonucleotides. *Gene* **50**: 313–320

Waldmann V and Rabes HM (1997) Absence of $G_s\alpha$ gene mutations in childhood thyroid tumours after Chernobyl in contrast to sporadic adult thyroid neoplasia. *Cancer Res* **57**: 2358–2361

Williams ED (1995) Mechanisms and pathogenesis of thyroid cancer in animals and man. *Mutat Res* **333**: 123–129

Wright PA, Lemoine NR, Mayall ES, Wyllie FS, Hughes D, Williams ED and Wynford-Thomas D (1989) Papillary and follicular thyroid carcinomas show a different pattern of RAS oncogene mutation. *Br J Cancer* **60**: 576–577

Wright PA, Williams ED, Lemoine NR and Wynford-Thomas D (1991) Radiation-associated and 'spontaneous' human thyroid carcinomas show a different pattern of RAS oncogene mutation. *Oncogene* **6**: 471–473

Wynford-Thomas (1993) Molecular basis of epithelial tumorigenesis: the thyroid model. *Crit Rev Oncogen* **4**: 1–23

© Cancer Research Campaign 1998